# Supplementary material for: Resolving the full spectrum of human genome variation using Linked-Reads
Source: Genome Res. 2019 Apr;29(4):635–45. doi: 10.1101/gr.234443.118 (PMC6442396; doi:10.1101/gr.234443.118)
Supplement: Supplemental Material [file supp_gr.234443.118_Supplemental_longranger-2.2.zip › longranger-2.2.tar.bz2!/lr2.2-public/lib/stan/cmdstan/stan/doc/api/html/arg__fail_8hpp_source.html]

Stan: src/stan/gm/arguments/arg\_fail.hpp Source File


|  |  |
| --- | --- |
|  | Stan  2.5.0  probability, sampling & optimization |


- Main Page
- Related Pages
- Namespaces
- Classes
- Files

- File List
- File Members

All Classes Namespaces Files Functions Variables Typedefs Enumerations Enumerator Friends Macros Pages

- src
- stan
- gm
- arguments

arg\_fail.hpp

Go to the documentation of this file.

1 #ifndef STAN\_\_GM\_\_ARGUMENTS\_\_FAIL\_\_HPP

2 #define STAN\_\_GM\_\_ARGUMENTS\_\_FAIL\_\_HPP

3

4 #include <stan/gm/arguments/unvalued\_argument.hpp>

5

6 namespace stan {

7

8  namespace gm {

9

10  class arg\_fail: public unvalued\_argument {

11

12  public:

13

14  arg\_fail() {

15

16  \_name = "fail";

17  \_description = "Dummy argument to induce failures for testing";

18

19  }

20

21  };

22

23  } // gm

24

25 } // stan

26

27 #endif

28

stan::gm::arg\_fail::arg\_fail

arg\_fail()

**Definition:** arg\_fail.hpp:14

unvalued\_argument.hpp

stan::gm::unvalued\_argument

**Definition:** unvalued\_argument.hpp:12

stan::gm::argument::\_name

std::string \_name

**Definition:** argument.hpp:76

stan::gm::arg\_fail

**Definition:** arg\_fail.hpp:10

stan::gm::argument::\_description

std::string \_description

**Definition:** argument.hpp:77


---

[ Stan Home Page ]

*© 2011–2014,
Stan Development Team.*
